# Typhoid Conjugate Vaccine: An Urgent Tool to Combat Typhoid and Tackle Antimicrobial Resistance

**DOI:** 10.1093/infdis/jiab443

**Published:** 2021-09-16

**Authors:** Samir K Saha, Nazifa Tabassum, Senjuti Saha

**Affiliations:** 1 Child Health Research Foundation, Dhaka, Bangladesh; 2 Department of Microbiology, Dhaka Shishu Hospital, Bangladesh Institute of Child Health, Dhaka, Bangladesh

**Keywords:** typhoid, vaccine, enteric fever, antimicrobial resistance

## Abstract

Typhoid is endemic in many countries in South Asia and sub-Saharan Africa. The high burden of this age-old, preventable disease exacerbates constraints on the health systems of these countries. Currently, most patients are treated effectively in the community or outpatient departments, but with rising antimicrobial resistance and the dearth of novel antimicrobials in the horizon, we risk losing our primary defense against typhoid. Extensively drug-resistant *Salmonella* Typhi is spreading, and azithromycin is the last oral drug to continue treating typhoid in the community. With increasing azithromycin resistance, emergence of pan-oral drug resistant *Salmonella* Typhi is imminent. The high burden of typhoid is also an underlying cause of the unnecessary use of antimicrobials. In addition to implementing water sanitation and hygiene interventions to prevent typhoid, it is imperative to rapidly roll out typhoid conjugate vaccines in endemic countries. This will not only reduce the burden of typhoid but will also help interrupt the trend of increasing antimicrobial resistance.

*Salmonella enterica serovar* Typhi, the causative agent of the age-old disease typhoid, is estimated to be responsible for 10.9 million illnesses and 116 800 deaths annually, on a global scale [1]. More than 80% of the total burden of typhoid rests on low- and middle-income countries (LMICs) in South Asia and sub-Saharan Africa. A human-restricted pathogen, *Salmonella* Typhi is transmitted by contaminated food and water, and the primary methods for controlling the burden of typhoid are improvements in water, sanitation, and hygiene, along with vaccination.

In many endemic countries, *Salmonella* Typhi is the predominant cause of bacterial bloodstream infections in children over the age of 2 months and can constitute up to two-thirds of all bacterial isolates [2]. Hence, typhoid poses a substantial burden on the overall health systems of these countries. In such resource-constrained settings, many patients with severe and potentially fatal diseases requiring medical care, like meningitis and neonatal sepsis, are refused hospitalization because beds are not available [3]. Physicians in the outpatient departments (OPDs) are able to spend less than a minute with each patient, adversely affecting patients’ overall health [4]. The high burden of this preventable disease exacerbates the constraints on these already struggling health systems.

## THE GROWING CRISIS OF ANTIMICROBIAL RESISTANCE

Currently, typhoid fever can be managed very effectively with the use of antimicrobials. Before the advent of antibiotics, the mortality rates related to typhoid fever exceeded 30% in several areas, but drugs such as chloramphenicol reduced this rate to <1% [5]. In endemic areas, majority of typhoid cases (both confirmed and suspected) are treated empirically with oral antimicrobials in OPDs and community clinics, or at pharmacies.

Concerningly, however, *Salmonella* Typhi has exhibited resistance to every widely used antimicrobial. Resistance to the 3 first-line drugs (ampicillin, chloramphenicol, and cotrimoxazole), defined as multidrug resistance, was widespread by the early 1980s [6]. Consequently, primary treatment shifted to fluoroquinolones, but by 2015, >90% of isolates were nonsusceptible to fluoroquinolones [6]. Ceftriaxone and azithromycin were left as the last 2 oral antibiotics for treatment of typhoid fever.

The increasing reports of antimicrobial resistance (AMR) has led to the increasing use of the last lines of antimicrobials for empirical treatment in hospitals, OPDs, and communities. In Bangladesh, for example, third-generation cephalosporins are the primary drug of choice for treatment of confirmed and suspected typhoid fever. As most endemic countries lack the facility or the resources to conduct blood culture and antimicrobial susceptibility testing, for each confirmed case of typhoid fever, 3–25 patients without a confirmed detection of *Salmonella* Typhi are treated with antimicrobials [7]. As a result, the regional presence of typhoid fever often leads to overtreatment of all suspected typhoid fever cases with unnecessary antimicrobials, thus indirectly driving the increasing AMR demonstrated in *Salmonella* Typhi and potentially other pathogenic bacteria. In recent years, many studies have depicted a decline in resistance to 3 of the first-line drugs, but treatment strategies have not been updated accordingly [8, 9].

The selective pressure due to widespread use of last-generation antimicrobials has led to growing concerns regarding untreatable typhoid fever. Indeed, an outbreak caused by an extensively drug-resistant (XDR) strain of *Salmonella* Typhi was identified in Pakistan in 2016; XDR *Salmonella* Typhi is resistant to chloramphenicol, ampicillin, trimethoprim-sulfamethoxazole, streptomycin, fluoroquinolones, and third-generation cephalosporins. Within 4 years of its detection, XDR *Salmonella* Typhi constituted >80% of the entire *Salmonella* Typhi population in Pakistan [10], and it has been detected in at least 10 countries [9]. For patients with uncomplicated XDR typhoid, azithromycin is the last option for an oral antimicrobial. But with increasing use of azithromycin in South Asia, azithromycin resistance is also on the rise.

To date, no XDR isolate has been identified as also azithromycin resistant, but recent studies have shown that azithromycin resistance can be caused by a single nonsynonymous point mutation, which can occur spontaneously [11, 12]. The rise of pan-oral drug-resistant (PoDR) Typhi may only be a matter of time [13]. The emergence and spread of PoDR *Salmonella* Typhi could pose serious threats to the health systems of LMICs, where typhoid is endemic, as typhoid patients would require hospitalization to be treated with injectable antibiotics like meropenem. This would cause an overwhelming increase in demand for hospital beds, which the resource-constrained settings may struggle to meet.

## VACCINES TO TACKLE TYPHOID BURDEN AND AMR

As therapeutic options dwindle, it is imperative to control typhoid aggressively through preventive measures and interrupt this trend of rising AMR. Improvements in water, sanitation, and hygiene are important steps for the prevention and control of typhoid, and Sustainable Development Goal 6, which targets universal and equitable access to safe and affordable drinking water, is projected to have positive implications in the control of typhoid fever [14]. However, this will require substantial multisectoral investments, infrastructural changes, and long-term commitments.

While such changes are gradually implemented, vaccines must be used to bring about rapid changes, protect vulnerable communities from typhoid fever, and reduce the global burden of this neglected disease. Although several types of typhoid vaccines are available, the World Health Organization (WHO) recommends the use of typhoid conjugate vaccines (TCVs) in routine immunization programs in typhoid-endemic countries [15]. Typhoid was previously thought to be prevalent in older children, but many studies have shown that it is prevalent in children <2 years of age; hence, TCVs will be absolutely necessary to fight this rising crisis [16, 17].

The first TCV to achieve WHO prequalification, in January 2018, was Typbar TCV, manufactured by Bharat Biotech, India [18]. This opened the door for global procurement and support from Gavi, the Vaccine Alliance [19]. A randomized clinical trial of Typbar TCV, in children between 9 months and 15 years of age in Nepal, showed an efficacy of 82% [20]. Similar results were observed in a cluster-randomized trial of Typbar TCV in Bangladesh, which showed an efficacy of 85% among infants and children aged 9 months to 16 years [21]. The study also found no serious vaccine-related adverse events. Along the same lines, recent data from Pakistan demonstrated that this vaccine has an efficacy of 95% against all culture-confirmed *Salmonella* Typhi, increasing to 97% against XDR *Salmonella* Typhi strains [22].

These results illustrate that typhoid can be effectively prevented in children in endemic settings. In December 2020, to ensure adequate supply and a greater variety of products and manufacturers, WHO granted prequalification of a second TCV, TYPHIBEV, manufactured by Biological E in India [19]. Several other TCVs are in various stages of development.

In response to the high burden of typhoid and widespread transmission of the XDR *Salmonella* Typhi strain, Pakistan became the first country to introduce Typbar TCV into their routine immunization program in 2019 [22]. Pakistan has since vaccinated >20 million children [23], and preliminary reports suggest a substantial reduction in typhoid cases following TCV introduction [24]. This was followed by approval for introduction of TCVs in Liberia and Zimbabwe [23]. In India, the municipal authorities in Navi Mumbai made the decision to introduce the Typbar TCV based on observed high rates of typhoid fever [25].

Considering the burden of typhoid fever on health systems, TCV can also lead to multiple indirect effects. Any preventable disease that has a large footprint on the health system, such as bed usage and physician consultation time, exacerbates treatments and outcomes of other diseases in the context of reduced heath system capacity [26]. Therefore, TCV will not only decrease the burden of typhoid, but it is also likely to decrease the number of people who are refused admission owing to lack of hospital beds or denied sufficient consultation time because of the burden of typhoid.

TCVs can also provide us with an edge in the arms race against rising AMR. Previous studies have estimated that the introduction of the pneumococcal conjugate vaccine led to reduction in AMR infections [27, 28]. Similarly, considering that the high prevalence of confirmed and suspected typhoid cases is a primary driver of antimicrobial usage in many endemic countries, decrease in typhoid burden with TCVs is likely to reduce overall use of antimicrobials. In fact, WHO recommends prioritization of introduction of TCV in countries with a high burden of AMR *Salmonella* Typhi [15].

For countries where typhoid is endemic, Gavi has started accepting applications for support to LMICs implementing WHO-prequalified TCVs into their routine and catch-up immunization programs [29]. Given the urgency of the situation, it is imperative to initiate conversations among global and national partners, policy makers, and scientists regarding urgent vaccine acquisition and introduction in all endemic regions.

TCVs are an urgent tool for rapid reduction of typhoid burden in endemic regions, which in turn will decrease the pressure on the overall health system of resource-constrained settings. Wide rollout of the vaccines will also prevent the rise of PoDR *Salmonella* Typhi and help us combat the overall rising crisis of AMR.

## Note

***Potential conflicts of interest.*** All authors: No reported conflicts. All authors have submitted the ICMJE Form for Disclosure of Potential Conflicts of Interest. Conflicts that the editors consider relevant to the content of the manuscript have been disclosed.
